# Pain management protocol implementation and opioid consumption in critical care: an interrupted time series analysis

**DOI:** 10.5935/0103-507X.20190085

**Published:** 2019

**Authors:** Bruno Adler Maccagnan Pinheiro Besen, Antonio Paulo Nassar Júnior, Fábio Holanda Lacerda, Carla Marchini Dias da Silva, Vanessa Tota de Souza, Eliana Vieira do Nascimento Martins, Ana Tarina Alvarez Lopes, Carlos Eduardo Brandão, Lucas Fernandes de Oliveira

**Affiliations:** 1 Unidade de Terapia Intensiva, Hospital da Luz - São Paulo (SP), Brasil.; 2 Unidade de Terapia Intensiva Clínica, Disciplina de Emergências Clínicas, Hospital das Clínicas, Faculdade de Medicina, Universidade de São Paulo - São Paulo (SP), Brasil.; 3 Unidade de Terapia Intensiva, Hospital AC Camargo Cancer Center - São Paulo (SP), Brasil.

**Keywords:** Pain, Pain measurement, Analgesics, opioid/adverse effects, Dipyrone, Intensive care units

## Abstract

**Objective:**

To evaluate the impact of an opioid-sparing pain management protocol on overall opioid consumption and clinical outcomes.

**Methods:**

This was a single-center, quasi-experimental, retrospective, before and after cohort study. We used an interrupted time series to analyze changes in the levels and trends of the utilization of different analgesics. We used bivariate comparisons in the before and after cohorts as well as logistic regression and quantile regression for adjusted estimates.

**Results:**

We included 988 patients in the preintervention period and 1,838 in the postintervention period. Fentanyl consumption was slightly increasing before the intervention (β = 16; 95%CI 7 - 25; p = 0.002) but substantially decreased in level with the intervention (β = - 128; 95%CI -195 - -62; p = 0.001) and then progressively decreased (β = - 24; 95%CI -35 - -13; p < 0.001). There was an increasing trend in the utilization of dipyrone. The mechanical ventilation duration was significantly lower (median difference: - 1 day; 95%CI -1 - 0; p < 0.001), especially for patients who were mechanically ventilated for a longer time (50^th^ percentile difference: -0.78; 95%CI -1.51 - -0.05; p = 0.036; 75^th^ percentile difference: -2.23; 95%CI -3.47 - -0.98; p < 0.001).

**Conclusion:**

A pain management protocol could reduce the intensive care unit consumption of fentanyl. This strategy was associated with a shorter mechanical ventilation duration.

## INTRODUCTION

Unpleasant sensations are common in patients admitted to intensive care units (ICUs). Pain accounts for a substantial burden of symptoms, and its effects may be detrimental not only in the short term^(1)^ but also in the long term.^(2)^ The Society of Critical Care Medicine (SCCM) guidelines on pain recommend a proactive approach to pain management that includes (1) pain evaluation with validated scales; (2) opioids as a first line therapy for pain; and (3) multimodal analgesia to spare opioids in some scenarios.^(3)^

Pain management in the ICU may be difficult for some reasons. The pharmacokinetics of opioid and nonopioid analgesics are altered due to organ dysfunction;^(4)^ patients also frequently experience hemodynamic instability. This leads to the choice of fentanyl rather than morphine as the usual first-line opioid used in critical care due to its pharmacokinetic properties and hemodynamic stability.^(5,6)^

However, the use of opioids - and fentanyl in particular - is not without drawbacks. Excessive opioid use may lead to intoxication-like symptoms because fentanyl is subject to a high context-sensitive half-life when infused for prolonged periods.^(4,6)^ Some adverse effects may negatively impact mechanical ventilation (MV) weaning: (1) respiratory drive dysregulation leading to high tidal volumes and low respiratory rates;^(7)^ and (2) a reduced level of consciousness.^(8)^

Pain in critical care has further characteristics that may not benefit from the high-dose continuous infusions of opioids. Pain and discomfort are frequently related to mechanical reasons, such as fecal impaction, urinary retention, device traction and the patient's position on the bed, which are not treated adequately with and may even be worsened by opioids. Pain is also more intense during procedures,^(9)^ which could lead to a higher benefit of boluses of drugs instead of high-dose continuous infusions.

Therefore, our primary objective was, in a single intensive care unit, to implement a pain management routine and to assess its impact on overall fentanyl consumption. Furthermore, we evaluated routinely measured clinical outcomes to assess the potential benefits of an opioid-sparing strategy for pain management and the potential reduction in costs related to opioid consumption.

## METHODS

This was a single-center, quasi-experimental, retrospective, before and after cohort study. Consent to participate was waived by the Institutional Review Board (IRB) given the retrospective nature of the study (IRB approval: 1.700.252/CAAE: 58827116.0.0000.5533). This manuscript adheres to the STROBE guidelines.

The ICU at *Hospital da Luz* is a mixed ICU comprised of 20 beds. Unit staffing includes the following: one physician for five beds during the morning and one physician for every 10 beds during the afternoon, nightshifts and weekends; one nurse for every seven beds during the day and every 10 beds during night shifts; one nurse assistant for every two beds; and one physical/respiratory therapist for every 10 beds. Daily multidisciplinary rounds are performed to set daily goals of care.

Until September 2014, the pain management strategy for mechanically ventilated patients was based on high-concentration fentanyl infusions (50µg/mL), which is a common practice in Brazil. On October 2014, one of the authors implemented a new pain management protocol, which consisted of the following:

1. Systematic evaluation of pain with validated and standardized pain scales: a numerical rating scale for patients who were able to communicate and a Behavioral Pain Scale (BPS) for patients who could not be assessed otherwise.^(10,11)^
2. Regular use of dipyrone as an adjuvant for analgesia.
3. Use of diluted solutions of fentanyl (10µg/mL), starting at 10 - 20µg/hour when necessary, and using boluses (10 - 50µg) before painful procedures as necessary, such as tracheal suctioning.
4. Staff training about the equianalgesic doses of fentanyl and morphine (10µg fentanyl = 1mg morphine).

The study population used to evaluate the clinical outcomes comprised all patients admitted in the study period. We defined the "before" cohort as those patients admitted to the ICU from January 1, 2014, until September 30, 2014. We defined the "after" cohort as those patients admitted to the ICU from November 1, 2014, until December 31, 2015. We excluded patients admitted during October 2014 from the clinical outcomes analysis because it was the month of implementation of this ICU culture change, which could have had carry-over effects on the "after" cohort. We also excluded patients who were readmitted to the ICU from the clinical outcomes analysis. Given the retrospective nature of the study, there was no formal sample size calculation.

The primary outcome was the consumption of analgesics, which was analyzed in aggregate. The secondary outcomes were individually measured clinical outcomes and aggregated analgesic-related costs.

We retrieved variables related to monthly consumption of both opioid and nonopioid analgesics from the hospital database: intravenous (IV) morphine (2 and 10mg ampules), fentanyl (IV, 500µg ampules), tramadol (IV, 100mg ampules), dipyrone (IV, 1g ampules) and ketoprofen (IV, 100mg ampules). We built monthly rates with patient-days in the ICU and MV days as denominators. For this analysis, we did not exclude any ICU admissions and considered all patients to create the denominators (ICU and MV patient-days). Furthermore, we measured the costs related to each unit to grossly evaluate monthly costs related to analgesic consumption. We did not measure costs related to drug preparation, such as syringes, needles and dilution fluids.

We retrieved variables of all patients during the study periods from the ICU quality database (Epimed Monitor^®^), which was recorded by a trained nurse during the study period and routinely audited for its accuracy, and it has been described previously.^(12)^ The measured baseline variables included age, sex, (Simplified Acute Physiology score 3 (SAPS 3), Sequential Organ Failure Assessment (SOFA) score, premorbid functional status, Charlson comorbidity score and type of admission. Use of vasopressors, MV and renal replacement therapy were retrieved from the database (at 24 hours or any time during the ICU stay). The measured outcomes were ICU mortality, hospital mortality, ICU length-of-stay (LOS), hospital LOS, MV duration, use of parenteral nutrition at any time during the ICU stay (as a surrogate of severe gastrointestinal dysfunction) and renal replacement therapy after 24 hours of ICU stay (as a surrogate of potential nephrotoxicity related to dipyrone). Moreover, we retrieved the number of self-extubations from the adverse event reporting database.

### Statistical analysis

We analyzed the monthly consumption of analgesics with a time series analysis.^(13)^ We compared the mean rate of monthly consumption in the two study periods in a standard bivariate analysis with t-tests or Wilcoxon rank-sum tests, as appropriate. We built a segmented linear regression model to evaluate three different aspects of the time series:

y = β_0_ + β_1_ * *time* + β_2_ * *level* + β_3_ * (*time* * *intervention*); where:

- β_1_ = slope of the trend of utilization before the intervention (from January through October 2014).
- β_2_ = change in the level of utilization of analgesics when the intervention was implemented (October 2014).
- β_3_ = slope of the trend of utilization after the intervention (from November 2014 through December 2015).

We used the Prais-Winsten method to account for 1^st^ order autocorrelation in the primary analysis. Durbin-Watson statistics, Durbin's alternative tests and adjusted R^2^ were evaluated to assess model adequacy.

Clinical variables were first analyzed with a Fisher's exact test for categorical variables. Continuous variables were assessed for normality with the Shapiro-Wilk test and analyzed with unpaired t-tests or the Wilcoxon rank-sum test, as appropriate. To obtain a meaningful interpretation of variables with a skewed distribution, we calculated median differences with 95% confidence intervals (95%CI) obtained from the Hodges-Lehmann estimator. Second, to provide adjusted estimates, we evaluated binary outcomes (ICU mortality; hospital mortality; and RRT after 24 hours of ICU admission) through a multivariable logistic regression model adjusted for confounders at baseline. We built all models with available clinically relevant variables at baseline to adjust for confounding (SAPS 3, performance status, and the use of vasopressors at 24 hours and of MV at 24 hours). We used quantile regression (at percentiles of 25, 50 and 75) to assess the impact of the intervention period on the duration of MV because it was a highly skewed variable precluding linear regression analysis.^(14)^ Standard errors were estimated using 1,000 bootstrap replications, and we adjusted only for the SAPS 3 and performance status to allow convergence of the model. All adjusted analyses were complete cases since we had < 1% missing data. All analyses were performed at the 0.05 alpha level. We used Stata SE 14.2 to run all analyses.

## RESULTS

**Figure 1:**
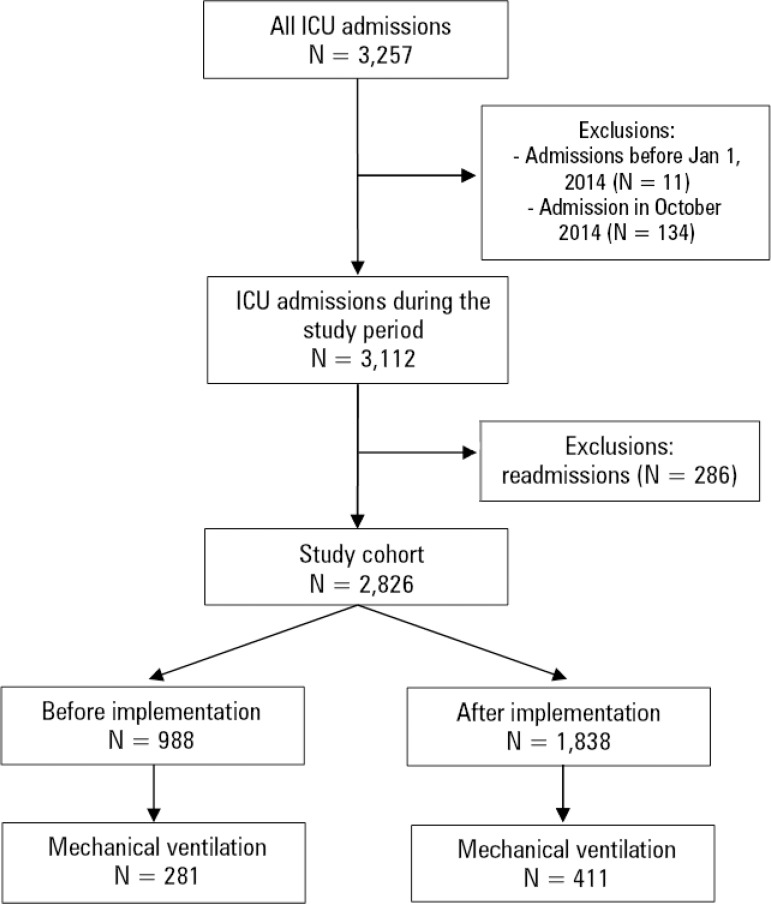
Flowchart of the study participants. ICU - intensive care unit.

During the study period, 3,257 patients were admitted. We excluded 134 patients who were admitted on October and 286 patients who were readmissions (Figure 1). We included 988 patients in the preintervention period and 1,838 in the postintervention period. Patients in the postintervention period were slightly older, more predominantly admitted for medical reasons, had slightly higher SAPS 3 scores, were less independent functionally and were less frequently mechanically ventilated than patients in the preintervention period (Table 1).

**Table 1:** Sample characteristics

Variable	Preintervention N = 988	Postintervention N = 1,838	p value
Age	56 (18)	58 (20)	0.012*
Male sex	437 (44.5)	770 (42.3)	0.259†
Admission type			< 0.00†
Medical	614 (62.2)	1,284 (69.9)	
Elective surgery	299 (30.3)	416 (22.6)	
Emergency surgery	75 (7.6)	138 (7.5)	
SAPS 3	44.2 (17.5)	45.5 (15.7)	0.045*
SOFA score	1 [0 - 4]	1 [0 - 4]	0.743§
Charlson comorbidity score	2 [0 - 3]	2 [0 - 3]	0.258§
Premorbid functional status			< 0.001†
Independent	892 (90.3)	1,559 (84.8)	
Partial assistance	44 (4.4)	117 (6.4)	
Bedridden	52 (5.3)	162 (8.8)	
Organ support			
Vasopressors	258 (26.1)	379 (20.6)	0.001†
Mechanical ventilation	281 (28.4)	411 (22.4)	< 0.001†
Renal replacement therapy	61 (6.2)	120 (6.5)	0.748†

SAPS - Simplified Acute Physiology Score; SOFA - Sequential Organ Failure Assessment during the first 24 hours of intensive care unit admission.

* t-test;

† Fisher exact test;

§ Wilcoxon rank-sum test. Results expressed as mean (standard deviation), n (%) or median [p25 - P75].

### Primary outcome - Time series analysis

**Figure 2:**
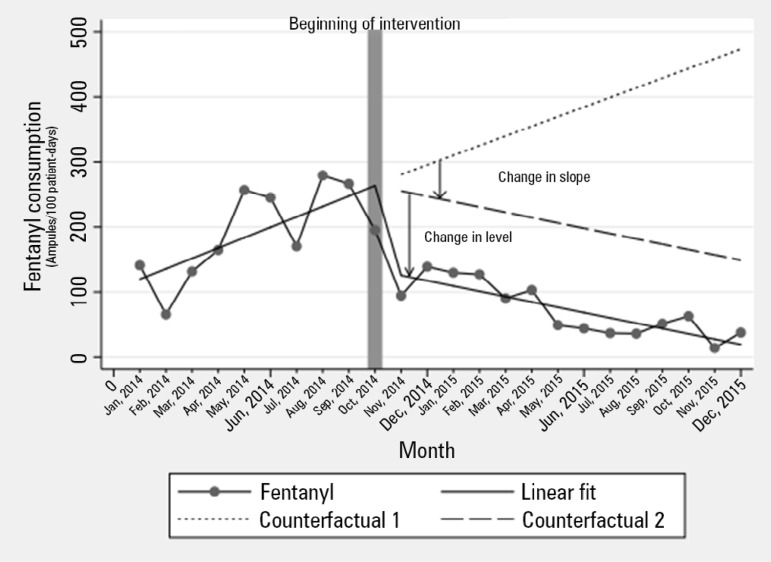
Monthly observed and predicted fentanyl consumption. Counterfactual 1 represents what would be the expected consumption of fentanyl if there was no difference in trend or level of fentanyl consumption. Counterfactual 2 represents the expected consumption of fentanyl if there were no differences in the level of fentanyl consumption. The predicted values are derived from the model presented in table 2.

Visual inspection of the time series plots (Figures 1S and 2S - Supplementary material) showed two major trends: an increase in the use of dipyrone and a reduction in the consumption of fentanyl. In the segmented regression analysis by ICU patient-days, fentanyl presented with an increasing trend in use before the intervention (β = 16; 95%CI 7 - 25; p = 0.002), which decreased in level (β = - 128; 95%CI -195 - -62; p = 0.001) and then in slope for a decreasing trend (β = - 24; 95%CI -35 - -13; p < 0.001) (Table 2, Figure 2). In the analysis by MV-days, the results were comparable.

**Table 2:** Interrupted time series analysis of the primarily measured analgesic consumption*

Variable	Trend before intervention (β1)			Change in level (β2)			Trend after intervention (β3)			Model adjusted R^2^
	Mean	95%CI	p value	Mean	95%CI	p value	Mean	95%CI	p value	
Fentanyl (ampules)										
Per month	74	28 - 119	0.003	-546	-878 - -213	0.003	-112	-167 - -58	< 0.001	0.70
Per 100 patient-days	16	7 - 25	0.002	-128	-195 - -62	0.001	-24	-35 - -13	< 0.001	0.76
Per 100 MV -patient-days	15.5	-0.1 - 31.1	0.051	-141	-257 - -25	0.020	-36	-53 - -18	< 0.001	0.80
Dipyrone (ampules)										
Per month	-6	-50 - 38	0.773	34	-294 - 361	0.833	102	51 - 154	< 0.001	0.86
Per 100 patient-days	-3	-10 - 4	0.373	6	-47 - 59	0.821	24	16 - 32	< 0.001	0.91
Per 100 MV-patient-days	-37	-97 - 24	0.220	-174	-623 - 274	0.427	225	156 - 293	< 0.001	0.91
Morphine equianalgesic dose (mg)†										
Per month	3,889	1,722 - 6,057	0.001	-28,277	-44,212 - -12,343	0.001	-5,798	-8,356 - -3,240	< 0.001	0.72
Per 100 patient-days	835	406 - 1,263	0.001	-6,666	-9,839 - -3,493	< 0.001	-1,234	-1,734 - -734	< 0.001	0.78
Per 100 MV-patient-days	823	30 - 1,615	0.043	-7,435	-13,316 - -1,553	0.016	-1,692	-2,587 - -796	0.001	0.76

* Adjustment for 1^st^ order autocorrelation with the Prais-Winsten method;

† Equianalgesic doses for 1mg of morphine. 95% CI - 95% confidence interval; MV - mechanical ventilation.

Dipyrone had no significant trend before the intervention, and the level did not change during the month of the intervention, but the slope after the intervention was significant and showed an increasing trend of utilization of this analgesic (Table 3).

**Table 3:** Clinical outcomes

Outcome	Preintervention N = 988	Postintervention N = 1,838	Effect estimate* (95%CI)	p value
ICU mortality, n(%)				
Crude	119 (12.0)	183 (9.9)	0.81 (0.63 - 1.03)	0.087†
Adjusted	-	-	0.92 (0.67 - 1.27)	0.612‡
Hospital mortality, n(%)				
Crude	200 (20.2)	334 (18.2)	0.87 (0.72 - 1.06)	0.180†
Adjusted	-	-	0.81 (0.63 - 1.04)	0.093‡
RRT after 24 hours, n(%)				
Crude	22 (2.2)	36 (1.9)	0.87 (0.51 - 1.50)	0.632†
Adjusted	-	-	0.95 (0.55 - 1.65)	0.859‡
Parenteral nutrition, n(%)				
Crude	11 (1.1)	19 (1.0)	0.93 (0.44 - 1.96)	0.844†
MV duration§				
Median [P25 - P75]	2 [1 - 6]	1 [0 - 4]	1 (0, 1)	< 0.001¶
Adjusted, P 25	-	-	- 0.19 (- 0.70 - 0.31)	0.454||
Adjusted, Median	-	-	- 0.78 (- 1.51 - - 0.05)	0.036||
Adjusted, P 75	-	-	- 2.23 (- 3.47 - - 0.98)	< 0.001||
ICU LOS				
Median [P25 - P75]	2 [1 - 4]	2 [1 - 3]	0 (0, 0)	0.002¶
Hospital LOS				
Median [P25 - P75]	8 [4 - 15]	7 [4 - 13]	0 (0, 1)	0.039¶

95%CI - 95% confidence interval; ICU - intensive care unit; LOS - length of stay; RRT - renal replacement therapy; MV - mechanical ventilation.

* Odds ratio for categorical variables; median differences for quantitative variables;

† Chi-squared test;

‡ Logistic regression model adjusted for SAPS 3, performance status, use of vasopressors in the first 24 hours and use of mechanical ventilation in the first 24 hours;

§ Analysis only in patients under mechanical ventilation (N (pre) = 281; N (post) = 411);

¶ Wilcoxon rank-sum test;

|| quantile regression adjusted for SAPS 3 and performance status with 1,000 bootstrap replications.

Among other analgesics, only 2mg morphine had an increasing trend of utilization after the intervention - change in slope (Table 1S - Supplementary material). Other analgesics had no difference in their consumption rates (Figure 1S - Supplementary material; Table 1S - Supplementary material). The results of equianalgesic doses of morphine (1mg) were driven mainly by fentanyl consumption, and the results were in line with this (Table 2).

### Secondary outcomes

Length of intensive care unit stay and hospital mortality were the same in the two time periods; use of renal replacement therapy and parenteral nutrition also did not differ between the two groups (Table 3). Mechanical ventilation duration was significantly lower in unadjusted analyses (median difference: -1 day; 95%CI -1 - 0; p < 0.001), as were the ICU and hospital lengths of stay (Table 3). The reduction in MV duration was not significant in percentile 25 (difference -0.19; 95% CI -0.69 - -0.31; p = 0.454), which represents patients with ≤ 1 day of MV. The reduction was significant at percentile 50 (difference: -0.78; 95%CI -1.51 - -0.05; p = 0.046), and it was more evident in percentile 75 (difference: -2.23; 95%CI -3.47 - -0.98; p < 0.001), which represents patients with > 4 days of MV (Table 3). There were 6/333 (0.018%) self-extubations in the preintervention period and 12/485 (0.025%) in the postintervention period (p value = 0.6313).

### Analgesic costs

There was a significant reduction in the costs of measured analgesic consumption per 100 ICU patient-days, from R$ 844,00 before the intervention to R$ 664,00 after the intervention (mean difference -180, 95%CI -350 - -11, p-value = 0.039; Table 2S - Supplementary material). This was driven mainly by reductions in fentanyl-related costs (mean difference: - R$ 363,00).

## DISCUSSION

Our study shows that an intervention aiming to improve pain management can reduce ICU opioid utilization to approximately 40% of a previous baseline level and may lead to a sustained trend of lower utilization both in the short term and medium term (up to more than one year after the intervention). This occurred in parallel to increases in the use of dipyrone, without any detrimental effects observed in clinical outcome analyses in the before and after cohorts. The intervention also significantly reduced analgesic-related monthly costs and was associated with a median reduction in MV duration of 1 day: this effect was more prominent in patients who spent a larger number of days under artificial ventilation.

Although pain assessment is strongly recommended in guidelines,^(3)^ its widespread adoption is not universal. In a recent cross-sectional study of 45 ICUs in the United Kingdom, physicians did not document pain assessment in almost two-thirds of the patients; nurses did not document pain assessment in 28.6% of the patients.^(15)^ Luetz et al. found better results in a European multinational survey: 81 out of 101 ICUs reported assessing pain, but only 24 used a scale validated for patients who were unable to communicate.^(16)^ A nationwide Dutch study confirmed these findings: a wide adoption of pain scales for patients able to communicate and a low use of behavioral pain scales.^(17)^

Previous studies have shown the efficacy of the systematic assessment of pain in critically ill patients. In a large cohort study, pain assessment was associated with a shorter duration of MV and reduced ICU length of stay.^(18)^ Before and after studies have confirmed these findings.^(19-21)^ Our study, with a similar design, also showed a reduced duration of MV, especially for those patients who received MV for longer durations, suggesting a dose-response effect plausibly explained by the high context-sensitive half-life of fentanyl.

The reduction in opioid use can be considered unexpected. While a study showed that better pain assessment increased fentanyl use per patient,^(21)^ others showed opposite results.^(19,20)^ Our findings are in accordance with the latter. Although it would be counterintuitive to make an association between better pain assessment and decreased use of opioids, there are some possible reasons for this result. First, routine pain management strategies focus on pain assessment. Therefore, as opportunities for assessment increase, dose reevaluation also increases. In the period before our strategy implementation, physicians and nurses were initiating fentanyl infusion in high doses, as recommended in previous guidelines,^(5)^ and without standardized periodic reevaluations. This approach could mean using fentanyl as a sedative, which could only potentiate fentanyl prolonged effects.^(4,8)^ Previous studies on "analgesia first" strategies used very low doses of opioids. For example, in a landmark study of "no sedation" in patients undergoing MV, analgesia was maintained with 2.5 - 5mg of morphine as needed.^(22)^ In a Brazilian study comparing daily interruption of sedatives and a sedation protocol in critically ill patients on MV, patients used only a median of 300mcg of fentanyl per day in the sedation protocol group.^(23)^ Second, a multimodal analgesia approach could spare opioid consumption. Dipyrone use increased after the implementation of our pain management approach. In the previously mentioned cohort study, Payen et al. demonstrated that nonopioids were used more frequently when pain was systematically assessed.^(18)^ Many studies in critically ill patients show that the use of nonopioid analgesics decreases the use of opioids without differences in pain scores;^(24)^ enables lighter sedation levels;^(25)^ and reduces the time to extubation.^(26)^ Our study is the first to show that dipyrone may be a reasonable nonopioid to be used in critically ill patients. At the very least, it seems to be as good as paracetamol for use in a multimodal approach to spare opioids.^(27)^ A third reason that we believe may have had a role in reduced opioid consumption is the use of a diluted solution of fentanyl: a continuous infusion of 10mL/h of this dilution represents 100µg/h of fentanyl, while the previous infusion represents 500µg/h of fentanyl. Although physicians and nurses may know the actual concentration of fentanyl in each solution, cognitive biases regarding the infusion speed may lead to unwanted 2- to 3-fold higher doses of the drug.

This study has some limitations. First, although we performed adjusted analyses to compare the two periods, we could not adjust for all possible confounders; other variations in care and secular trends may also have contributed to the observed results - especially MV duration and ICU length of stay - since the ICU medical team had changed at the time of the implementation of the protocol, although nurses and physical therapy teams remained unchanged throughout the study period. Nevertheless, the substantial reduction in opioid consumption is a clinically significant result that leads to a substantial reduction in unnecessary spending and may have an impact on clinical outcomes. Second, we do not have pain measurements available for the purposes of this study, and therefore, we cannot prove that patients had adequate pain control. However, all the ICU staff were trained to evaluate and treat pain properly, with special attention given to mechanical causes of pain (such as fecal impaction or urinary retention - best treated with mechanical maneuvers) and to preprocedural analgesia. Furthermore, this does not invalidate the findings of the intervention in the aggregate measures. Third, our results are from a single-center study and may not be generalizable, although these findings could help others scrutinize their pain management protocols, which can have an impact on clinical outcomes. Fourth, one major side effect of opioids that we could not address with our methodology was the development of paralytic ileum, constipation and reduced tolerance to enteral nutrition; further studies will be necessary to address these issues.

## CONCLUSION

An intensive care unit pain management protocol characterized by routine pain assessment, increased use of dipyrone and use of a diluted solution of fentanyl substantially reduced the intensive care unit consumption of fentanyl. This strategy was also associated with a shorter mechanical ventilation duration.
