# Supplementary material for: Pain management protocol implementation and opioid consumption in critical care: an interrupted time series analysis
Source: Rev Bras Ter Intensiva. 2019 Oct-Dec;31(4):447–55. doi: 10.5935/0103-507X.20190085 (PMC7009002; doi:10.5935/0103-507X.20190085)
Supplement: Supplementary file 1 [file rbti-31-04-0447-suppl1.pdf]

Pain management protocol implementation and opioid consumption in critical care: an interrupted time series analysis

Implantação de um protocolo de manejo de dor e redução do consumo de opioides na unidade de terapia intensiva: análise de série temporal interrompida

Bruno Adler Maccagnan Pinheiro Besen[1,2], Antonio Paulo Nassar Júnior[3], Fábio Holanda Lacerda[1,2,3], Carla Marchini Dias da Silva[1,3], Vanessa Tota de Souza[1], Eliana Vieira do Nascimento Martins[1], Ana Tarina Alvarez Lopes[1], Carlos Eduardo Brandão[1,3], Lucas Fernandes de Oliveira[1,3]

STROBE Statement - Checklist of items

	Item No	Recommendation	Page No
**Title and abstract**	1	(a) Indicate the study's design with a commonly used term in the title or the abstract	447
		(b) Provide in the abstract an informative and balanced summary of what was done and what was found	447
**Introduction**			
Background/rationale	2	Explain the scientific background and rationale for the investigation being reported	447, 448
Objectives	3	State specific objectives, including any prespecified hypotheses	448
**Methods**			
Study design	4	Present key elements of study design early in the paper	448, 449
Setting	5	Describe the setting, locations, and relevant dates, including periods of recruitment, exposure, follow-up, and data collection	448, 449
Participants	6	(a) Give the eligibility criteria and the sources and methods of selection of participants. Describe methods of follow-up	448, 449
		(b) For matched studies, give matching criteria and number of exposed and unexposed	NA
Variables	7	Clearly define all outcomes, exposures, predictors, potential confounders, and effect modifiers. Give diagnostic criteria, if applicable	448, 449
Data sources/measurement	8*	For each variable of interest, give sources of data and details of methods of assessment (measurement). Describe comparability of assessment methods if there is more than one group	449
Bias	9	Describe any efforts to address potential sources of bias	449
Study size	10	Explain how the study size was arrived at	448
Quantitative variables	11	Explain how quantitative variables were handled in the analyses. If applicable, describe which groupings were chosen and why	449
Statistical methods	12	(a) Describe all statistical methods, including those used to control for confounding	449
		(b) Describe any methods used to examine subgroups and interactions	NA
		(c) Explain how missing data were addressed	449
		(d) If applicable, explain how loss to follow-up was addressed	NA
		(e) Describe any sensitivity analyses	449
**Results**			
Participants	13*	(a) Report numbers of individuals at each stage of study—e.g., numbers potentially eligible, examined for eligibility, confirmed eligible, included in the study, completing follow-up, and analyzed	449
		(b) Give reasons for non-participation at each stage	449, Figure 1
		(c) Consider use of a flow diagram	Figure 1

Continue...

... continuation

	Item No	Recommendation	Page No
Descriptive data	14*	(a) Give characteristics of study participants (e.g., demographic, clinical, social) and information on exposures and potential confounders	449, Table 1
		(b) Indicate number of participants with missing data for each variable of interest	NA
		(c) Summarize follow-up time (e.g., average and total amount)	449, 450, Table 3
Outcome data	15*	Report numbers of outcome events or summary measures over time	450, 451, Table 3
Main results	16	(a) Give unadjusted estimates and, if applicable, confounder-adjusted estimates and their precision (e.g., 95% confidence interval). Make clear which confounders were adjusted for and why they were included	450, 451, Table 3
		(b) Report category boundaries when continuous variables were categorized	450, 451, Table 3
		(c) If relevant, consider translating estimates of relative risk into absolute risk for a meaningful time period	NA
Other analyses	17	Report other analyses done—e.g., analyses of subgroups and interactions, and sensitivity analyses	450, 451, Supplementary material
**Discussion**			
Key results	18	Summarize key results with reference to study objectives	452
Limitations	19	Discuss limitations of the study, taking into account sources of potential bias or imprecision. Discuss both direction and magnitude of any potential bias	453
Interpretation	20	Give a cautious overall interpretation of results considering objectives, limitations, multiplicity of analyses, results from similar studies, and other relevant evidence	452, 453
Generalizability	21	Discuss the generalizability (external validity) of the study results	453
**Other information**			
Funding	22	Give the source of funding and the role of the funders for the present study and, if applicable, for the original study on which the present article is based	447

* Give information separately for exposed and unexposed groups. NA - not applicable. Note: An Explanation and Elaboration article discusses each checklist item and gives methodological background and published examples of transparent reporting. The STROBE checklist is best used in conjunction with this article (freely available on the Web sites of PLoS Medicine at http://www.plosmedicine.org/, Annals of Internal Medicine at http://www.annals.org/, and Epidemiology at http://www.epidem.com/). Information on the STROBE Initiative is available at http://www.strobe-statement.org.

Table 1S - Segmented linear regression analysis of other measured analgesic consumption

Variable	Trend before intervention (β1)			Change in level (β2)			Trend after intervention (β3)			Model adjusted R^2
	Mean	95%CI	p value	Mean	95%CI	p value	Mean	95%CI	p value	
Morphine 2 mg (ampules)										
Per month	1	-8 - 10	0.853	-40	-107 - 26	0.218	14	4 - 25	0.010	0.70
Per 100 patient-days	0.1	-1.7 - 1.9	0.877	-9	-23 - 4	0.172	3	1 - 5	0.007	0.71
Per 100 MV-patient-days	-1	-9 - 7	0.742	-56	-114 - 2	0.057	25	15 - 34	< 0.001	0.88
Morphine 10mg (ampules)										
Per month	17	3 - 31	0.021	-68	-172 - 37	0.191	-16	-32.5 - 0.5	0.056	0.14
Per 100 patient-days	3.6	0.3 - 6.9	0.035	-16	-40 - 8	0.178	-3.3	-7.1 - 0.6	0.091	0.09
Per 100 MV-patient-days	4	-12 - 20	0.627	-14	-132 - 103	0.801	4	-14 - 23	0.637	0.18
Tramadol 100mg (ampules)										
Per month	5	-4 -14	0.281	23	-46 - 92	0.498	-11	-22 - 0.1	0.052	0.11
Per 100 patient-days	0.6	-1.4 - 2.6	0.520	3.7	-11.2 - 18.7	0.607	-1.8	-4.2 - 0.5	0.118	0.09
Per 100 MV-patient-days	-9	-24 - 6	0.216	75	-36 - 187	0.173	14	-3 - 32	0.107	0.23
Ketoprofen 100mg (ampules)										
Per month	0.13	-1.82 - 2.09	0.888	0.07	-14.39 - 14.52	0.993	-0.74	-3.02 - 1.54	0.505	0
Per 100 patient-days	-0.01	-0.47 - 0.44	0.950	-0.17	-3.54 - 3.2	0.918	-0.1	-0.63 - 0.43	0.698	0
Per 100 MV-patient-days	-1	-3.09 - 1.09	0.329	5.48	-9.99 - 20.96	0.469	1.15	-1.29 - 3.59	0.337	0.08

95%CI - 95% confidence interval; MV - mechanical ventilation.

Table 2S - Mean monthly cost (in Brazilian reais - R$) of analgesic drugs before and after the intervention

Variable	Before	After	Mean difference (95% CI)	p value*
Fentanyl	584 (212)	221 (124)	- 363 (- 526 - -202)	< 0.001
Morphine (2mg)	47 (23)	117 (73)	70 (26 - 114)	0.004
Morphine (10mg)	65 (53)	70 (27)	5 (-34 - 45)	0.767
Tramadol	38 (7)	36 (9)	- 2.1 (-8,7 - 4.4)	0.504
Dipyrone	101 (16)	213 (71)	112 (70 - 154)	< 0.001
Ketoprofen	10 (5)	7 (5)	- 3 (-10.8 - 1.6)	0.195
Total	844 (233)	664 (79)	- 180 (-350 - -11)	0.039

95%CI - 95% confidence interval. * All p values were calculated with an unpaired t test accounting for unequal variances. The Wilcoxon rank-sum test results agreed with all t tests at the 0.05 alpha level.

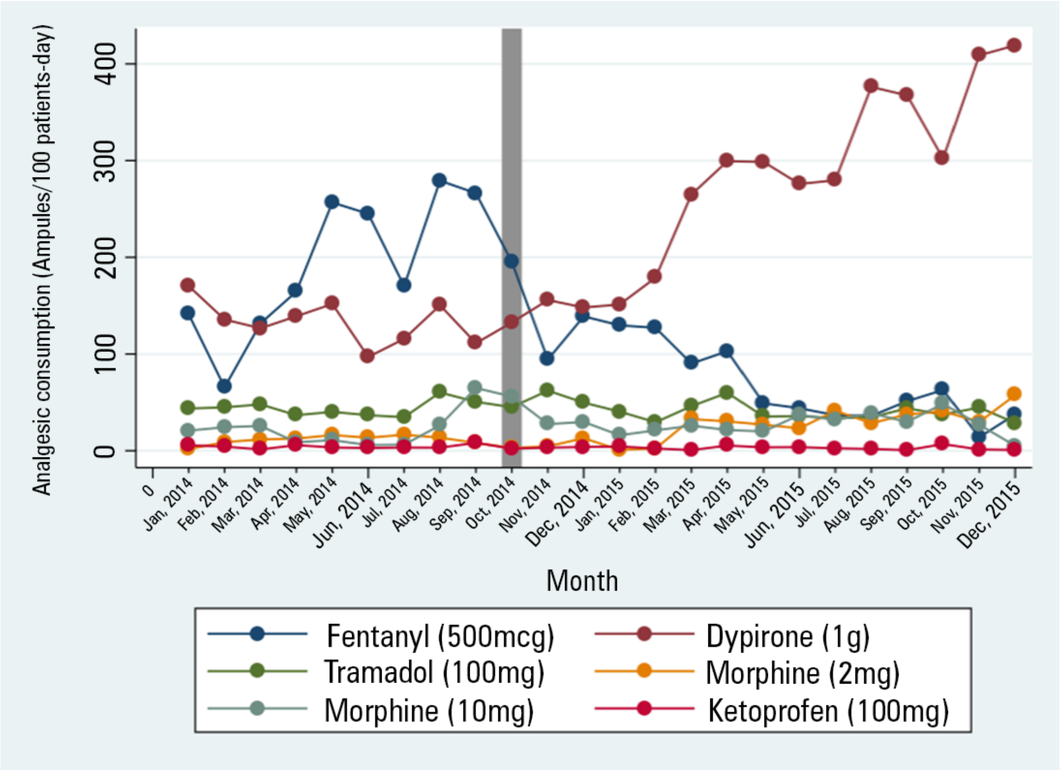

Figure 1S - Analgesic consumption per month.

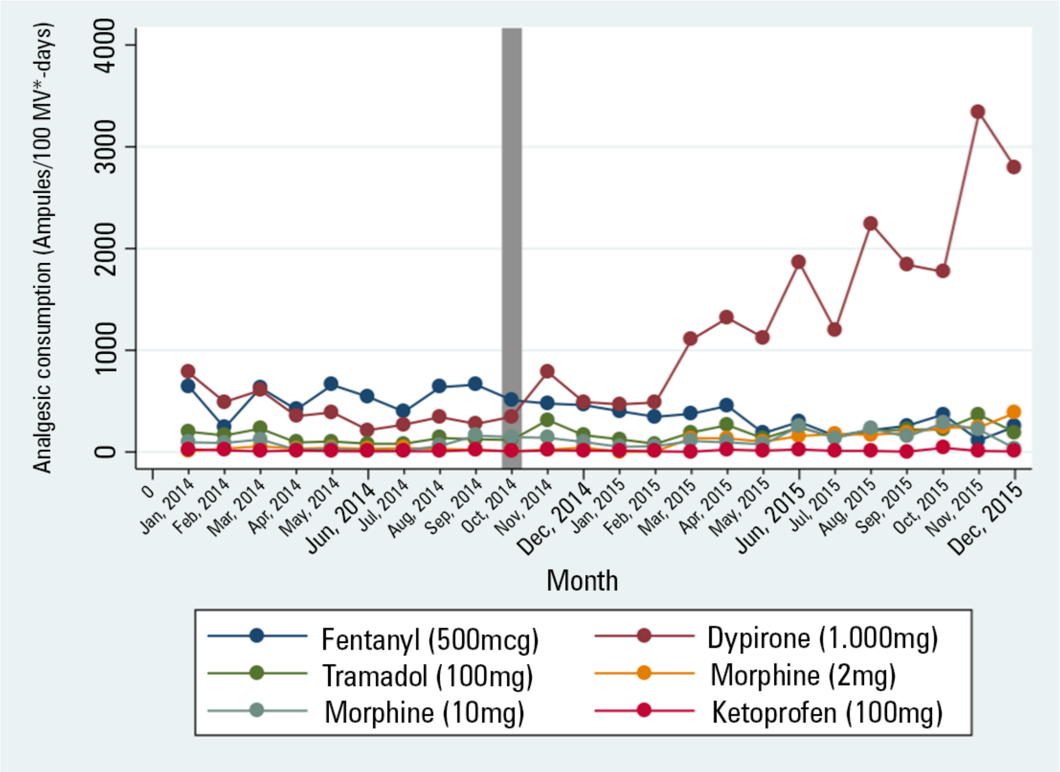

Figure 2S - Analgesic consumption per month. MV - mechanical ventilation.
